# Remimazolam and Its Place in the Current Landscape of Procedural Sedation and General Anesthesia

**DOI:** 10.3390/jcm13154362

**Published:** 2024-07-25

**Authors:** Matthew Brohan, Janette Brohan, Basavana Goudra

**Affiliations:** 1 Resident in Anesthesiology, School of Medicine, University College Cork, T12 K8AF Cork, Ireland; mpbrohan@gmail.com; 2 Cork University Hospital, T12 DC4A Cork, Ireland; janettebrohan@gmail.com; 3 Honickman Center, Department of Anesthesiology, Sidney Kimmel Medical College, Jefferson Health, Philadelphia, PA 19107, USA

**Keywords:** remimazolam, pediatric, sedation, endoscopy, bronchoscopy, general anesthesia, dental, electrophysiology, intensive care

## Abstract

Remimazolam was derived from its parent compound by adding an ester linkage into its structure so that the drug becomes a substrate for ester metabolism. As a result, it undergoes organ-independent ester hydrolysis, although the clinical benefits in terms of shorter recovery are not uniformly observed in clinical practice. Remimazolam is mainly tested in procedural sedation. In comparison to propofol, the current gold standard for procedural sedation, its proposed attractiveness is shorter wake-up times and a clear-headed recovery. Its clear advantages over propofol are better hemodynamic stability, lack of pain on injection and availability of a reversal agent in the form of flumazenil. Data on patient and proceduralist satisfaction are lacking. Remimazolam is also used for induction and maintenance of general anesthesia in Japan (where it is approved for this purpose). In this scenario, it is not clear if it can achieve the same degree of lack of recall as propofol. The use of remimazolam in obstetrics, pediatrics and high-risk populations is an emerging area.

## 1. Introduction

Remimazolam is a novel ester-benzodiazepine sedative first synthesized in the 1990s that has been approved by the FDA for clinical use since 2020 [1]. While the FDA has only approved it for the induction and maintenance of procedural sedation in adults, it is also approved for general anesthesia in Japan and South Korea [2]. Remimazolam is available in two salt forms—remimazolam besylate and remimazolam tosylate [1]. It adopts many of the characteristics of its parent compounds, midazolam (pharmacodynamic) and remifentanil (pharmacokinetic), and as a result, it provides sedation with a quicker rate of onset and offset than midazolam [3]. This unique pharmacokinetic profile was established by incorporating a carboxyl ester linkage in the midazolam molecule so that it became a substrate for esterase metabolism that affords exclusive elimination advantage. The end product is an inactive carboxy acid metabolite (CNS 7054) [4,5]. Remimazolam acts on the GABA (γ-aminobutyric acid)-A receptor benzodiazepine (BZD) binding site and potentiates the effects of GABA. It induces a conformational change in the GABA-A receptor (GABAAR), which facilitates the binding of GABA, the primary inhibitory neurotransmitter in the central nervous system. As GABA binds to the receptor, it opens a ligand-gated ion channel, which results in an influx of many ions, primarily chloride ions. This hyperpolarizes the neural cell membrane, reducing membrane reactivity and inducing its sedative effect [1]. Benzodiazepine receptors have several isoforms (subclassifications), which accounts for the wide-ranging effects of benzodiazepines. It is thought that the BZ1 receptor is responsible for the sedative and amnesic effects, while the BZ2 subtype contributes to the anxiolytic and myorelaxant effects seen in benzodiazepines. Research is in progress to synthesize a selective benzodiazepine1 (BZ1) receptor agonist [6]. Given the wide range of effects seen with benzodiazepines, they can be clinically unpredictable. This establishes the need for softer agents, such as remimazolam, that can be titrated to its desired effect. Soft pharmacology refers to any compound that is rapidly metabolized into inactive metabolites after the desired therapeutic effect is achieved. 

Remimazolam is metabolized by tissue carboxylesterases, primarily hepatic tissue carboxylesterases, and is excreted in the urine as an inactive metabolite via hydroxylation and glucuronidation [2,7]. The primary tissue esterase responsible for the metabolism of remimazolam is carboxylesterase 1 (CES1). While CES1 is found in high concentrations in the liver, it is also expressed in the lungs and gallbladder. CES1 is implicated in the metabolism of many other drugs, such as clopidogrel and methylphenidate. While CES1 is expressed at its highest concentration in liver hepatocytes, it is thought that the non-hepatic metabolism of remimazolam is still significant [4]. 

The pharmacokinetic profile of remimazolam contrasts with that of its parent molecule, midazolam. Midazolam is metabolized through a hepatic route and excreted in the urine as 1-hydroxy-midazolam [8]. When compared to midazolam, the route of metabolism and excretion of remimazolam is not affected by drugs that induce or inhibit the Cytochrome P450 enzyme family [3,9]. Its metabolism is not dependent on the liver and, as such, it is thought that it may find particular use in those with mild liver impairment [4,10]. The pharmacokinetics of remimazolam appears to be linear with the dose, and systemic clearance could be as high as three times that of midazolam. Considering that its metabolite (excreted renally) is biochemically inactive, it may be safer in individuals with renal impairment [11]. The affinity of its carboxyl metabolite CNS 7054 for the BZD binding site is 300–400 times weaker than remimazolam and, as a result, is found to have no significant off-site effects [4].

Comparing the pharmacokinetics of remimazolam in a variety of different populations, the following appears to be accurate. The pharmacokinetics of remimazolam are largely similar across the age groups, both pediatric and elderly. Patients with mild hepatic impairment display similar pharmacokinetic profiles to those without liver disease [5]. This does, however, appear to contrast with populations with severe liver impairment, who were found to have reduced drug clearance than those with less severe liver impairment [5]. When compared to midazolam, remimazolam was found to have a more rapid onset and offset in its hypnotic effect at recovery time points of 10 and 40 min in phase 1 trials [12,13]. As with other benzodiazepines, the hypnotic effect of remimazolam is fully reversed with flumazenil, although after prolonged infusions, re-sedation or recurrent respiratory depression is possible [6,14,15]. 

## 2. Current Uses of Remimazolam

### 2.1. Gastrointestinal Endoscopy

The current standard for sedation in patients undergoing GI endoscopic procedures across much of the USA and Europe seems to be propofol. While in the USA, it is exclusively administered by anesthesia providers (anesthesiologists and nurse anesthetists), in Europe, nurses administer propofol under the supervision of the endoscopists performing the procedure. The merits and drawbacks of both approaches and the relative popularity of propofol across the world are discussed extensively in the literature [16,17,18,19,20,21,22,23,24,25]. The biggest impetus to finding a replacement for propofol comes from the cost (the cost of the anesthesia provider being the largest component), evolving insurance coverage issues, shortage of anesthesia providers and higher risk of complications. However, the high degree of both patient and endoscopist satisfaction is beyond any debate [17]. In addition, the increased efficiency may balance the additional cost [26,27,28,29]. Nevertheless, constant efforts to find a replacement for anesthesia provider-administered propofol have yielded little progress [30,31,32,33,34,35,36,37,38].

Due to its rapid onset and offset, remimazolam has a unique role in procedural sedation and the potential to replace propofol in some situations, such as uncomplicated screening colonoscopy. It is advantageous to have a sedative with quick onset and a short recovery time to shorten turnover times. Many studies have explored the feasibility of employing remimazolam as a sole agent or along with a short-acting opioid. Recent studies have also compared it with the current gold standard, propofol.

Very early studies on the utility of remimazolam in GI endoscopy sedation were hardly convincing. Even though the onset of action was shorter than its parent compound, midazolam, the offset of its clinical activity was barely better than propofol [39]. However, a chief observed advantage was that, in comparison to propofol, remimazolam was found to induce sedation with lower rates of associated blood pressure lability and respiratory depression. Most significantly, in this phase 1b dose-finding study of multiple doses of remimazolam in volunteers undergoing colonoscopy, as many as 56% of patients could not be adequately sedated, even after escalating the dose to 0.2 mg per Kg of body weight. Inadequate sedation was also responsible for incomplete colonoscopy in 11/44 subjects in another study [14].

Nonetheless, in a more recent large multicentric randomized controlled trial (461 randomized patients in 12 U.S. sites), incomplete colonoscopy due to inadequate sedation was less than 2% [40]. The investigators also found that the remimazolam group had an expedited recovery period, required less fentanyl, and felt “back to normal” much sooner than the midazolam and placebo groups. This may, in turn, have an economic benefit.

In a small single-center prospective randomized controlled trial involving 82 elderly patients, Jian Guo et al. had a success rate of 100% (successful completion of the procedure in all patients). In addition, remimazolam was associated with less frequent hemodynamic instability and respiratory depression [41]. They concluded by stating that remimazolam is safe and effective for GI endoscopy sedation in elderly patients with the added benefit of reduced hemodynamic events and respiratory depression. A major drawback of their study is the administration of propofol as an intermittent bolus, which is bound to increase the stated adverse events, especially in the elderly. In contemporary US practice, propofol is typically administered as a bolus followed by an infusion. Any hypotension is easily treatable with vasopressors. In yet another large phase 3 trial (Shao-Hui Chen et al.), remimazolam was nearly as good (non-inferior) as propofol [42]. The authors recruited a total of 384 patients scheduled to undergo upper gastrointestinal endoscopy at 17 centers between September 2017 and November 2017. A success rate of 97.34% (vs. 100.00% in the propofol group) with remimazolam is excellent. Although the safety of remimazolam was exceptional, there was no mention of patient and endoscopist satisfaction. Patients’ expectations are likely to be different in many Asian countries, where most studies have been performed. Unsedated colonoscopy [43,44,45] is performed widely and accepted by the local population in many Asian countries. 

A meta-analysis comparing three different randomized controlled trials on the use of remimazolam in patients undergoing colonoscopy found that the use of remimazolam was associated with less frequent top-up doses and lesser need for rescue medication in patients undergoing colonoscopy when compared to patients treated with midazolam [7,46]. Another meta-analysis examining hypotension in patients who underwent sedation for colonoscopy found that those sedated with propofol had much higher rates of hypotension than those who received remimazolam, RR 2.15 [1.61–2.87] [8,47]. 

A small but significant number of breastfeeding mothers present for GI endoscopic procedures, such as for inflammatory bowel disease. Unlike propofol, it is recommended that nursing mothers pump and discard breast milk for 5 h, even after a single dose [48].

In conclusion, depending on the patient population and the local culture, remimazolam can be effectively and safely administered to achieve adequate depth of sedation for GI endoscopic procedures with a very high degree of success. In many regions (from where the effectiveness data have come), especially in some Asian countries, endoscopists routinely perform these procedures with no or minimal sedation. These results need to be replicated in Western populations for their wider acceptance. Large studies are limited to colonoscopies, and data on the potential use of remimazolam in procedures such as EUS and ERCP are not available. 

### 2.2. Bronchoscopy

Remimazolam has been used as a sedative agent in the setting of bronchoscopy. Due to its rapid onset and offset of action, it was hypothesized that remimazolam would be an effective sedative for bronchoscopy. It is observed above that, as it pertains to GI endoscopy, several randomized controlled trials have indicated that remimazolam has a proven efficacy and safety profile when compared to midazolam [9,49]. In comparison, the availability of data is limited with regard to bronchoscopy.

In a prospective randomized controlled trial, remimazolam was seen to have a quicker onset of sedation, stronger safety profile and shorter neuropsychiatric recovery period (in comparison to midazolam) [10,50]. Considering that the investigators used flumazenil to reverse the effects of remimazolam, the two groups (remimazolam-flumazenil versus propofol) are not comparable. Surprisingly, contrary to most published evidence, the authors found that there were no significant differences in hemodynamic fluctuations or adverse events between the two groups. One would have expected greater hypotension and bradycardia in the propofol group. Nevertheless, it is incredible that the authors performed highly stimulating procedures such as rigid bronchoscopy with remimazolam. The administration of oxycodone, remifentanil and rocuronium along with high-frequency jet ventilation has clearly facilitated the process. As a result, the role of remimazolam in this study was as an alternative to propofol for providing hypnosis in the setting of general anesthesia.

Remimazolam was also compared with placebo and midazolam for moderate sedation during flexible bronchoscopy [51]. In this prospective, double-blind, randomized, multicenter, parallel-group trial performed across 30 US sites, the end points were safety and efficacy. As to be expected, like many studies discussed above in the section on GI endoscopy, their exploratory analysis demonstrated a shorter onset of action and faster neuropsychiatric recovery for remimazolam in comparison to midazolam. In another single-center, randomized controlled study that compared the safety and efficacy of remimazolam with those of midazolam for flexible bronchoscopy, patients receiving midazolam required more frequent reversal with flumazenil. Apart from being safe and effective, remimazolam exhibited a shorter onset time and faster neuropsychiatric recovery than midazolam [52]. Other investigators, including a meta-analysis, came to similar conclusions [53,54].

Lastly, Chen et al. compared remimazolam with dexmedetomidine for awake (sedated) tracheal intubation by flexible bronchoscopy. In this randomized, double-blind, controlled trial, they had equal success rates, good intubation conditions, and only minor respiratory depression. However, remimazolam had the added benefit of shorter intubation time, higher incidence of anterograde amnesia, and the ability to be antagonized by a specific antagonist [55]. 

### 2.3. Other Non-Operating Room Procedures

Sidhant Kalsotra et al. discussed sedation with remimazolam as a primary agent in three patients undergoing cardiac catheterization [56]. All patients had significant comorbidity involving pacemaker or conduction abnormalities. Both propofol and dexmedetomidine adversely impact inotropic, chronotropic or dromotropic function, and remimazolam has potential superiority in this area. The authors successfully completed catheterization without impact on hemodynamic or conduction function. Although all three patients were administered a bolus of remimazolam followed by infusion, several other sedative agents, such as midazolam, fentanyl, ketamine and propofol, were also administered. 

In a pilot study, Rudi Swart et al. reported their experience in sedating 25 adult subjects who received remimazolam and alfentanil for dental procedures [57]. Both patients and clinicians reported high satisfaction. 

### 2.4. General Anesthesia

Another potential role for remimazolam is in the induction and maintenance of general anesthesia. 

In a multicenter, single-blind, randomized, parallel-group, phase IIb/III trial, Matsuyuki Doi et al. examined three different doses of remimazolam for the induction of general anesthesia as compared to the propofol control [11,58]. At doses of 0.2 mg/kg, 0.3 mg/kg and 0.4 mg/kg, successful induction was 89%, 94% and 100%, respectively. In comparison with propofol, the success rate was 100%. The primary end point was the absence of intraoperative awakening/recall, the absence of a need for rescue sedatives, and the absence of body movements. The rates of hypotension in the first two dose groups were significantly lower than the propofol group, while they were similar in the highest dose group. A benefit of remimazolam groups was the absence of any injection site pain, while this was a common adverse effect seen in the propofol group (27%). 

In another study, the ED_50_ and ED_90_ for remimazolam to achieve loss of responsiveness within two minutes were 0.07 mg/kg/min (90% CI: 0.05, 0.09 mg/kg/min) and 0.10 mg/kg/min (90% CI: 0.10, 0.15 mg/kg/min), respectively [59]. At these rates, vital signs were stable, and no patients required inotropes/vasopressors. The authors concluded that general anesthesia may be induced at an infusion rate of 0.10 mg/kg/min within two minutes. In the absence of a continued infuser, 0.2 mg/kg may be used as an adequate bolus dose for induction. At a dose of 0.4 mg/kg, the incidence of hypotension is similar to a propofol bolus of 2 mg/kg [58,60]. 

A recent meta-analysis compared remimazolam and propofol for the induction and maintenance of general anesthesia [61]. In their analysis, Ko CC et al. included eight studies from 2008 to 2022. The results showed that remimazolam as an induction agent was associated with lower rates of post-induction hypotension, similar anesthetic efficacy and no injection site pain. Remimazolam was, however, associated with a lighter depth of anesthesia according to the bispectral index and a longer time to loss of consciousness when compared to propofol. Post-operative nausea and vomiting, time to eye-opening and extubation time were not seen to be significantly different between the remimazolam and propofol groups.

In conclusion, remimazolam is an attractive induction agent with many benefits in relation to propofol. These include better hemodynamic stability, minimal or no pain on injection, and availability of a reversal. However, the downside is slower induction rates, lighter levels of anesthesia and slower wake-up times if employed for the induction and maintenance of anesthesia. A case report recounted a 71-year-old man who was scheduled for a robotic-assisted laparoscopic radical prostatectomy and needed flumazenil at emergence. Despite maintaining intraoperative bispectral index scores of 30–50, he experienced re-sedation before finally awakening on post-operative day 2. Nevertheless, adequate anesthesia depth may not be obtained even with high doses, requiring propofol [15]. At the time of writing, data were not available regarding the incidence of awareness under anesthesia.

A study by Shimamoto et al. explored factors associated with delayed extubation following induction and maintenance with remimazolam [62]. They found that BMI greater than 22, age greater than 79 and plasma albumin concentrations of less than 3.6 g/dL were associated with a prolonged time to extubation. While these data are from a single-center study on a small patient cohort (n = 65), it reveals potential predictors of prolonged time to extubation in patients under general anesthesia with remimazolam.

## 3. Emerging Opportunities for Remimazolam

### 3.1. Obstetrics

Remimazolam does not have a well-established role in the obstetric setting. A retrospective cohort study examined the use of an infusion of remimazolam versus propofol for general anesthesia directly following cesarean section delivery [63]. This was a small cohort of 51 patients who were divided across a remimazolam and a propofol arm. The primary outcome of this study was the number of uterotonic medications required in the intrapartum period. Secondary outcomes included estimated blood loss and length of hospital stay. While this study is a small cohort, its findings are novel and appear to demonstrate that there is no significant difference in the number of uterotonic agents required across groups and in the estimated blood loss. The remimazolam group may have a reduced length of stay when compared to the propofol group; however, this is based on a marginally non-significant linear regression result, −0.5 days (95% CI, −1.02 to 0.02). This difference may, however, hold a clinical relevance. 

No information is currently available in the literature regarding the safety of remimazolam in patients who wish to breastfeed [48]. Based on its short duration of action, the current recommendation appears to be for breastfeeding patients to pump and discard milk for the first five hours following any dose of remimazolam. 

### 3.2. Pediatrics

While remimazolam is not currently licensed by the FDA for the pediatric population, there has been some research into the future application of remimazolam in this group [12,64]. The pharmacokinetic profile of remimazolam in the pediatric population appears to be similar to that in the adult population [65]. In this study, 24 pediatric patients undergoing surgeries of >2 h duration were induced and maintained on fentanyl, rocuronium and propofol. They were administered a continuous infusion of remimazolam for sixty minutes. Blood samples from these patients were then taken at various time points to examine the plasma concentrations of both remimazolam and its metabolite, CNS 7054. It was found that remimazolam demonstrated a three-compartment pharmacokinetic profile, while its metabolite demonstrated a two-compartment pharmacokinetic profile. In pediatric patients, remimazolam’s pharmacokinetic profile appears to have a high clearance rate with a context-specific half-life. 

Remimazolam may hold a role in reducing post-op delirium in children following ENT surgery. A randomized controlled study examined 104 children following tonsillectomy and adenectomy. These patients were induced and maintained using inhaled sevoflurane before receiving remimazolam towards the end of their procedure. It was found that patients administered with remimazolam at the end of their procedure had a significant reduction in the rate of emergence delirium when compared to a control [66]. It was also notable that when compared to a saline control, these patients had no difference in rates of post-operative pain scores, PACU length of stay or behavioral changes. The use of remimazolam in ASA 1–3 adult patients undergoing elective orthopedic surgery was associated with a 15.6% incidence of post-operative delirium (compared with 12.4% in the propofol group) [67]. While studying the effect of remimazolam on post-operative delirium in children aged 1–6 years, Yu-Hang Cai et al. found an incidence of 5% with remimazolam infusion, while it was 7.7% with a single bolus. The placebo group (saline) experienced a delirium rate of 32.5%. Similarly, while only 2 patients needed propofol to treat delirium in both remimazolam groups, that number was 10 in the saline group [68]. All children underwent laparoscopic surgery. 

Further research into the clinical application of remimazolam in the pediatric population is warranted, and there have been several research protocols proposed for this research [69,70]. 

### 3.3. Intensive Care Unit

Two studies have examined the prolonged use of remimazolam in the intensive care unit (ICU) setting for the sedation of patients on mechanical ventilation. The first, by Tang et al., was a randomized controlled trial comparing a cohort of patients treated with remimazolam besylate and propofol [71]. The primary outcome was sedation range on the Richmond Agitation and Sedation (RAAS) scale, with secondary outcomes including ventilator-free days at day 7, the length of ICU stay and 28-day mortality. When comparing the two groups, the remimazolam group was found to be non-inferior in terms of primary and secondary outcomes. 

The second study, by Yao et al., was a prospective study that compared patients who received remimazolam tosylate to those who received either propofol or midazolam for sedation for mechanical ventilation in the ICU setting [72]. Their primary outcome was ICU mortality, and secondary outcomes included laboratory tests, adverse events and the length of ICU stay. There was no significant difference between groups in terms of mortality, ICU length of stay, adverse events and RAAS scores. The remimazolam group demonstrated less variability in heart rate, lactate, bicarbonate, arterial blood gases and kidney and liver function when compared to the propofol/midazolam group.

### 3.4. High-Risk Groups

Given its unique pharmacokinetic profile, it is thought that remimazolam may have a particular role as a sedative agent in certain high-risk groups. 

Remimazolam has been shown to have a favorable safety profile in elderly populations when compared to propofol. In elderly patients undergoing hip replacement surgery, those receiving remimazolam had lower rates of hemodynamic compromise, cognitive dysfunction and respiratory depression as compared to propofol [13,73]. This is similar to the case in elderly patients undergoing gastrointestinal endoscopy [41]. A randomized controlled trial by Liu et al. demonstrated that the change in mean arterial pressure in elderly patients undergoing aortic valve replacement was lower in the group treated with remimazolam than in those treated with propofol [74]. This supports the evidence first seen in a retrospective study by Miyoshi et al. that reached the same conclusion [75]. They found that elderly patients treated with remimazolam require reduced vasopressor support to maintain hemodynamic stability. The post-operative recovery period for elderly populations treated with remimazolam also appears to be less complicated than those treated with other sedative agents from several perspectives. It appears to be associated with more expeditious neuropsychiatric recovery; however, this warrants further investigation.

The role of remimazolam as a sedative agent in patients with advanced renal failure is not well established. A single case study on its use in a hemodialysis patient suggests that remimazolam may have a role in these patients due to its organ-independent metabolism. In this case study involving an elderly hemodialysis patient undergoing general anesthesia with the use of remimazolam and remifentanil, it was noted that there were no major adverse effects, emergence from anesthesia was rapid and flumazenil was not required [76]. As previously mentioned, the metabolite of remimazolam has a very low affinity to the GABA-AR BZD binding site and no significant side effects; as a result, it may find use in this patient cohort. This is a clear opportunity for further investigation.

There is little research into the use of remimazolam in those with advanced liver disease. A single case study by Ushida et al. discussed the use of remimazolam in the treatment of a patient with Child–Pugh grade C liver cirrhosis and found that in this patient, emergence from anesthesia was prolonged [77]. This is an area of study that warrants further investigation, as advanced liver disease is not uncommon, and its perioperative management is challenging. 

## 4. Conclusions

Remimazolam is a novel benzodiazepine drug that has seen application in many settings, from endoscopy to general anesthesia. It has a proven safety profile and efficacy in a variety of situations. While its use in some situations is limited, in the future, there may be additional established roles for remimazolam in pediatric and obstetric anesthesia practice, in the intensive care setting and in the management of high-risk patients. These uses do, however, require further studies. A summary of the pros and cons discussed here is provided in Table 1.

The literature is inundated with reviews discussing the role and utility of remimazolam in the field of sedation and anesthesia [78,79,80]. Such reviews are mainly focused on basic pharmacology, with limited discussion on its clinical application, especially in non-operating room procedures. The current review fills this void, and practicing physicians, both anesthesia providers and proceduralists, are likely to find it very useful. 

## Figures and Tables

**Table 1:** Pros and cons of remimazolam.

Focus	Pros	Cons
Pharmacology	Unique ester-dependent hydrolysis, largely immune to any specific organ dysfunction, predictable metabolism, relatively faster recovery, especially during shorter procedures; availability of a reversal agent (flumazenil)	Similar mechanism of action (to its parent compound, midazolam) with similar onset of action
Comparison to propofol, the current gold standard for deep sedation	Better hemodynamic stability causing less bradycardia and hypotension, lesser need for vasopressors	Need for flumazenil reversal
GI endoscopy	Reduced blood pressure lability and respiratory depression (compared to propofol). Faster recovery (compared to midazolam); safe to use in elderly; effective for sedated (awake) endotracheal intubation	Higher failure rate (inability to complete the procedure) and lower patient and endoscopist satisfaction when compared to propofol; breastfeeding mothers need to pump and discard breast milk for 5 h (unlike propofol, where the mothers can immediately resume breastfeeding) [48]
Bronchoscopy	Quicker onset of sedation, stronger safety profile and shorter neuropsychiatric recovery period	Limited data to support its use, need for flumazenil
General anesthesia	No pain on injection, lower rates of hypotension	Limited experience, mainly from Japan, higher risk of awareness, no studies comparing with propofol in terms of recall, similar rates of hypotension (as propofol) at doses that provide 100% induction success, slower induction rates, re-sedation, no data on awareness, prolonged extubation, especially in elderly.
ICU sedation	Similar ventilator-free days (as propofol) at day 7, length of ICU stay and 28-day mortality (compared to propofol)	Very limited experience and data
Obstetrics	No difference in the need for uterotonic agents in patients receiving remimazolam for general anesthesia directly following cesarean section delivery	Mothers need to pump and discard breast milk for 5 h (unlike propofol, where the mothers can immediately start breastfeeding)
Pediatrics	Pharmacokinetics are similar to adults, potentially reduced risk of emergency delirium	No FDA approval for pediatric use
